# Epidemiology of *Taenia saginata* taeniosis/cysticercosis in the Russian Federation

**DOI:** 10.1186/s13071-018-3236-3

**Published:** 2018-12-14

**Authors:** Branko Bobić, Lian F. Thomas, Olgica Djurković Djaković, Brecht Devleesschauwer, Veronique Dermauw, Pierre Dorny, Uffe Christian Braae, Lucy Robertson, Anastasios Saratsis, Ramon Eichenberger, Paul R. Torgerson

**Affiliations:** 1 Centre of Excellence for Food and Vector-borne Zoonoses, Institute for Medical Research, University of Belgrade, Dr Subotića 4, Belgrade, 11000 Serbia; 2 Institute of Infection and Global Health, University of Liverpool, Liverpool, UK; 3 International Livestock Research Institute (ILRI), P.O. Box 30709, Nairobi, Kenya; 4 Department of Epidemiology and Public Health, Sciensano, 1050 Brussels, Belgium; 5 Department of Veterinary Public Health and Food Safety, Faculty of Veterinary Medicine, Ghent University, 9820 Merelbeke, Belgium; 6 Department of Biomedical Sciences, Institute of Tropical Medicine, 2000 Antwerp, Belgium; 7 Laboratory of Parasitology, Ghent University, 9820 Merelbeke, Belgium; 8 One Health Center for Zoonoses and Tropical Veterinary Medicine, Ross University School of Veterinary Medicine, Basseterre, Saint Kitts and Nevis; 9 Parasitology, Faculty of Veterinary Medicine, Norwegian University of Life Sciences, Oslo, Norway; 10 Veterinary Research Institute, Hellenic Agricultural Organisation Demeter, 57001 Thermi, Greece; 11 Centre for Biodiscovery and Molecular Development of Therapeutics, Australian Institute of Tropical Health and Medicine, James Cook University, QLD, Cairns, Australia; 12 Institute of Parasitology, Vetsuisse Faculty, University of Zurich, 8057 Zurich, Switzerland; 13 Section of Epidemiology, Vetsuisse Faculty, University of Zurich, 8057 Zurich, Switzerland

**Keywords:** *Taenia saginata*, Beef tapeworm, Taeniosis, Bovine cysticercosis, Russian Federation

## Abstract

**Background:**

Russia is traditionally an endemic area for *Taenia saginata* infection, where a programme for the prevention of infection has been implemented for sixty years. This paper aims, therefore, to review the recent epidemiology data of *Taenia saginata* infection in the Russian Federation.

**Methods:**

We undertook a systematic review of published and grey literature, and official data for information on the incidence, prevalence and distribution of *Taenia saginata* taeniosis and cysticercosis in the Russian Federation between 1st January 1991 and 31st December 2017.

**Results:**

From the 404 records returned by our search strategy, we identified 17 official county reports, 17 papers and one meeting abstract on the occurrence of taeniosis or cysticercosis from the Russian Federation, eligible for inclusion in this study. In the Russian Federation, *Taenia saginata* infection has been continuously present and notifiable in the study period between 1991–2016. In the same area, a continuous decrease in the incidence of human taeniosis cases was observed, from 1.4 to 0.04 cases per 100,000 inhabitants, as well as a reduction in the territory where the infection is reported. The prevalence of bovine cysticercosis, ranging between 0.1–19.0%, generally has a declining trend, especially after 2005.

**Conclusions:**

Importance of *Taenia saginata* infection as a medical and veterinary problem has been decreasing in the 21st century but it is still an infection with health and economic impact in the Russian Federation.

## Introduction

The Russian Federation (RF) spans about 17 million square kilometers and occupies more than one tenth of the Earth’s surface area. The Ural Mountains mark the traditional border between European and Asian Russia. In addition to the Ural Mountains, mountainous areas are present in far eastern Russia, while the rest of the country consists of wide plains with occasional low hills. All types of climate, except tropical, are present in the country, although winters are mostly long and cold, and summers are short. The northern parts of the country are covered by large pine forests, while the southern parts are covered by steppe. A small subtropical zone exists along the coast of the Black and Caspian seas. In addition to a major Slavic population, the RF is a multi-ethnic state with more than 100 ethnic groups and approximately 150 million inhabitants [1].

Traditionally, the RF is an endemic area for *Taenia saginata*. In the first half of the twentieth century, *T. saginata* infections were recognized as an important health and economic problem in the then Union of Soviet Socialist Republics (USSR). In the 1930’s, the need for systemic prevention was defined [2]. As a result, in the 1960’s a broad action to combat infection, that included epidemiological studies, health education, monitoring, the development of diagnostic capacity and therapy, and wastewater treatment, was implemented [3]. Although eradication of the infection as the definitive goal has never been achieved, the result of the aforementioned measures was a significant reduction in the incidence of taeniosis, from 58,400 cases in 1960 to 29,900 in 1965 (almost 50%) [4]. Half of these cases were reported from the territory of the then Russian Republic, the largest republic of the USSR, which today comprises practically the entire RF [5]. The decrease in the prevalence of *T. saginata* taeniosis continued over the following decades [6], until the dissolution of the USSR in 1991, when further monitoring moved to the jurisdiction of the newly born states.

In order to actively detect and prevent the spread of parasitic diseases, routine preventive examinations of employees in organizations whose activities are related to the production, storage, transportation and sale of food and drinking water, the upbringing and education of children, and community and household services are undertaken. Preventive measures for taeniosis include case reporting and an epidemiological study of the sources of all diagnosed cases and, of course, treatment under medical supervision [7–9]. Meat inspection by veterinarians at slaughterhouses as well as in slaughtering households, at the point of sale (markets, trades) and meat processing plants is obligatory. It includes visual inspection (with incisions in doubtful cases) of the tongue, external and internal masseter muscles with a mandatory 6–8 cuts and of the heart muscle along with several longitudinal and transversal cuts [10, 11]. Meat and meat products delivered for sale to markets, although previously inspected and branded (on the farm, slaughterhouse, meat processing plant, etc), are also subject to compulsory veterinary sampling and examination in laboratories [11].

When more than three cysticerci (more than five in case of reindeer) are found in sections of 40 cm^2^ of predilection muscles, the carcass is subject to technical disposal, while if less than three are present, the meat must be frozen. According to the safety regulations for the use of carcasses of infected cattle, they must be frozen until a temperature of -12 °C is reached in the thickest part of the meat (the temperature is measured in the hip muscle at a depth of 7–10 cm). If the temperature in the meat at the recommended depth is -6–9 °C, the carcass must be stored in the refrigerator for at least 24 hours [11]. Meat that underwent freezing treatment is used for further processing but not for sale at markets, and the use of disinfected meat in home-made minced meat, dried meat, sausages, smoked products, as well as other meat products and semi-finished products is officially prohibited. In case of detection of infected meat, after mandatory reporting, inspection of the farm, including staff and livestock examination, with additional disinfection measures is carried out if possible.

Changes in the political and, more importantly, in the economic life of the RF after 1991 have undoubtedly affected all parts of society, including the area of health and veterinary prevention. Our aim was to compile and analyze the epidemiological data for *T. saginata* infection in the RF by a systematic review of scientific and grey literature including official reports published between 1991–2016.

## Methods

### Study design

We conducted a systematic search and review of internationally and locally published sources of information on the epidemiology (occurrence, prevalence, incidence, age, gender and geographical distribution) of *T. saginata* infection in the human and animal populations in the RF, published between 1991–2017.

### Databases and other sources

For published data we searched both international and Russian databases. The former was done in the PubMed (http://www.ncbi.nlm.nih.gov/pubmed) and Web of Science (http://ipscience.thomsonreuters.com/product/web-of-science/) databases. The following search phrase was used: (cysticerci* OR *C. bovis* OR taenia* OR tenia*OR saginata OR taeniarhynchosi OR taeniid AND Russia*). Among Russian databases, we searched DVGMU Librar (Far East Medical State University Library, http://www.fesmu.ru/elib) and eLIBRARY.RU (Scientific electronic library, https://elibrary.ru/query_results.asp), using the following search phrase: Taeniarhynchus saginatus OR *Taenia saginata* OR тениаринхоз (*T saginata* taeniosis in Russian) финноз, цепень крупный рогатый скот, Бычий цепень (Russian expressions for *C. bovis* infection). For doctoral theses we searched international OpenGrey (www.opengrey.eu/) and in Russian Dissercat com (www.dissercat.com/search) database, using the same search phrases. Additionally, we investigated some of the basic historical references from the 1960’s that are cited in two publications [2, 27].

For official reports, epidemiological bulletins and normative documents, we searched the official websites of the Russian government services using the Russian search phrases. Also, we searched for relevant records in meeting proceedings of the European Network on Taeniosis/Cysticercosis - CYSTINET (COST Action TD1302).

Data on population statistics and breeding/slaughtering of cattle have been extracted from the reports of the Russian Federal State Statistics and the Food and Agriculture Organization of the United Nations (FAO).

### Selection criteria

Eligibility of the databases search results was first evaluated based on the title, then the abstract and finally, on the basis of the entire document, following the flow diagram of the search strategy steps recommended by PRISMA [12]. Thus, all references resulting from the database search were first screened, by titles, for duplicates and it was checked whether they were published in the period 1991–2017. Then the abstracts were screened for eligibility and were excluded based on the following criteria: (i) if the study referred to parasites other than *T. saginata*; (ii) if the study did not apply to the Russian Federation; (iii) if the data presented in the study did not refer to the period 1991–2017; (iv) if the data presented in the study were not connected to the epidemiological characteristics of *T. saginata* infection but rather focused on clinical features, therapy or parasite biology; and (v) if the study was just a general review of the topic, without original data. At the next step, the entire text of the manuscript was evaluated. Selected publications were additionally scrutinized and were excluded if only repeating epidemiological data published in official reports. Official reports we considered as eligible sources of data.

### Data extraction and generation

If data on prevalence have been provided in the selected literature and reports, they were directly used. If only the number of cases had been given, we calculated the prevalence using the number of inhabitants according to official census data for human taeniosis, or for cysticercosis in cattle the number of slaughtered cattle in that year according to official reports as the denominator. Although there is a chance of under-reporting in RF (due to unregistered household slaughters), the use of recorded number of slaughtered cattle as a denominator could not have a greater influence on the value of prevalence since mandatory veterinary control is generally performed for registered slaughtering. In the statistical analysis we used univariate analysis of variance.

## Results

### Search results

**Fig. 1:**
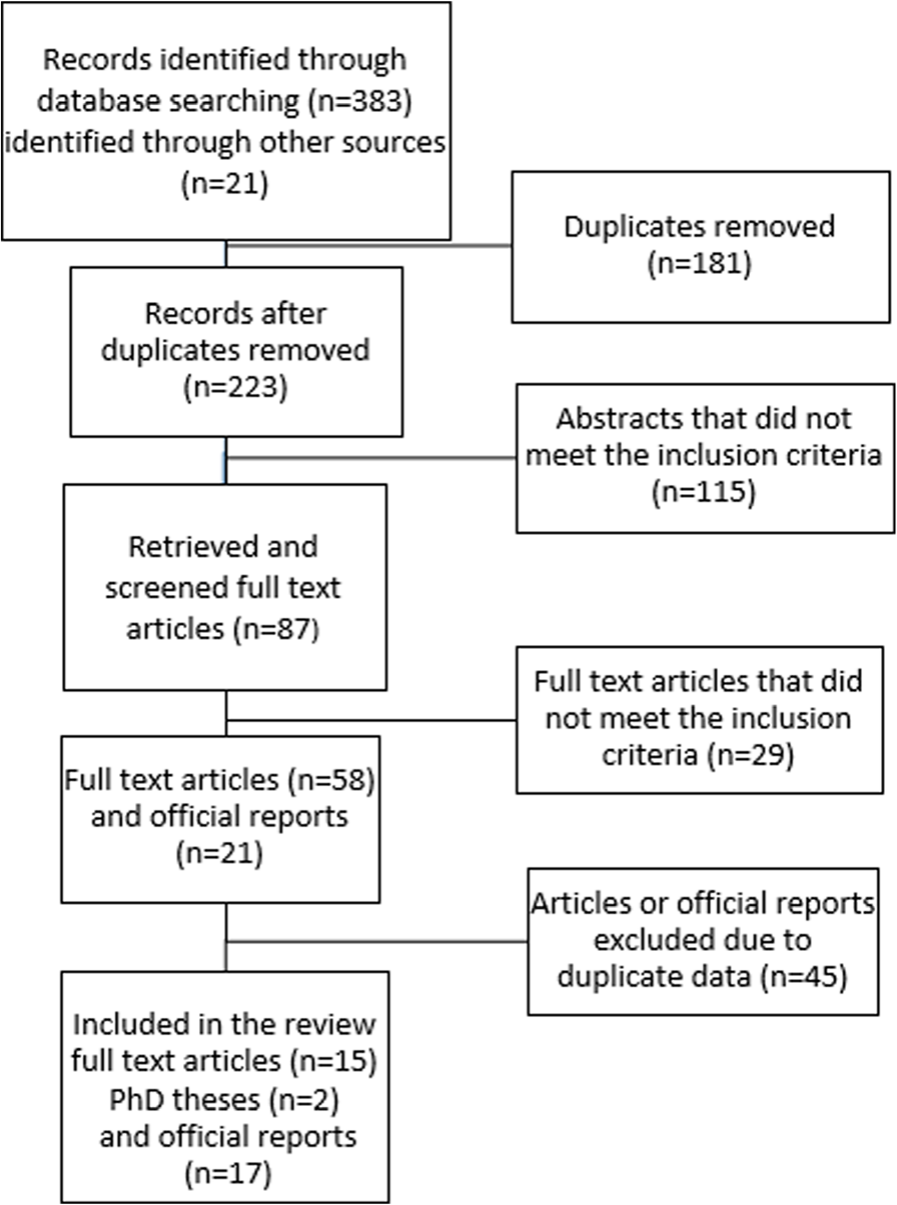
Flow diagram of the database searches

The database search results are outlined in the flow diagram (Fig. 1) A total of 34 relevant records were identified and included in the review; of these, 17 were official reports of RF ministries, 15 were peer reviewed papers and two were PhD theses. One additional meeting abstract was identified from proceedings of CYSTINET meeting. All eligible references were included in the analysis.

The *T. saginata* taeniosis prevalence values in humans have already been listed in the relevant selected documents and included as such in the results. Data on cattle infection included only the number of infected slaughtered cattle, so the prevalence had to be calculated first.

### Occurrence of *Taenia saginata* infection in humans

**Fig. 2:**
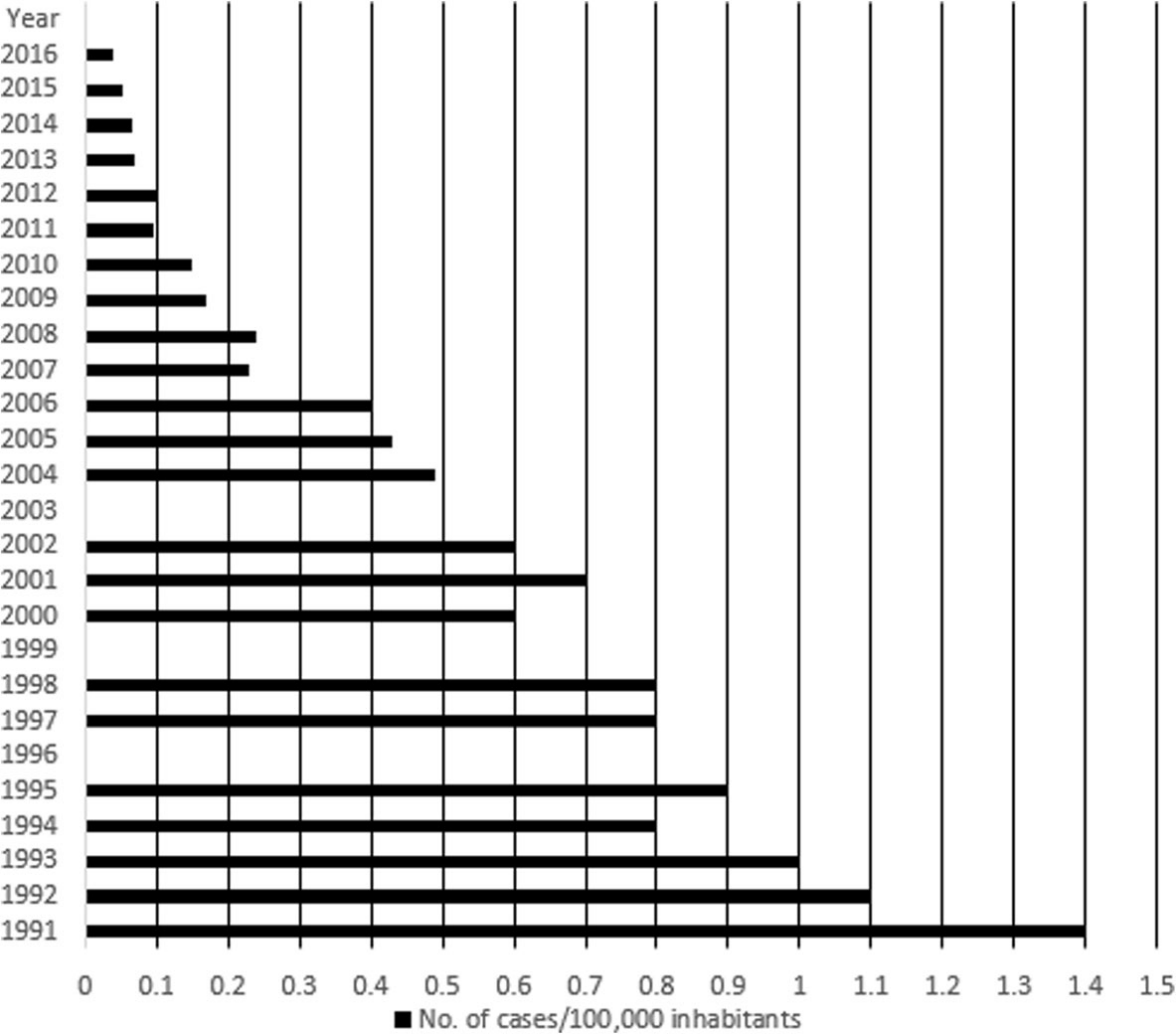
Number of cases *of Taenia saginata* taeniosis per 100,000 inhabitants officially reported in the Russian Federation (1991–2016) (data for 1996, 1999 and 2003 not available)

Between 1991–2016, according to official data [9, 13–17], more than 17,000 cases of *T. saginata* taeniosis were registered, with data not available for 1996, 1999 and 2003. The incidence significantly declined over the period of interest (*F*_(1,22)_= 35.465, *P* < 0.0001), from 1.4/100,000 in 1991 to 0.04/100,000 inhabitants in 2016 (Fig. 2). Of the reported cases, 5–18% of these were believed to be imported cases, with the majority occurring mostly in residents of the former USSR republics. The diagnostic method used for all reported human infections was microscopic examination of fecal samples.

**Fig. 3:**
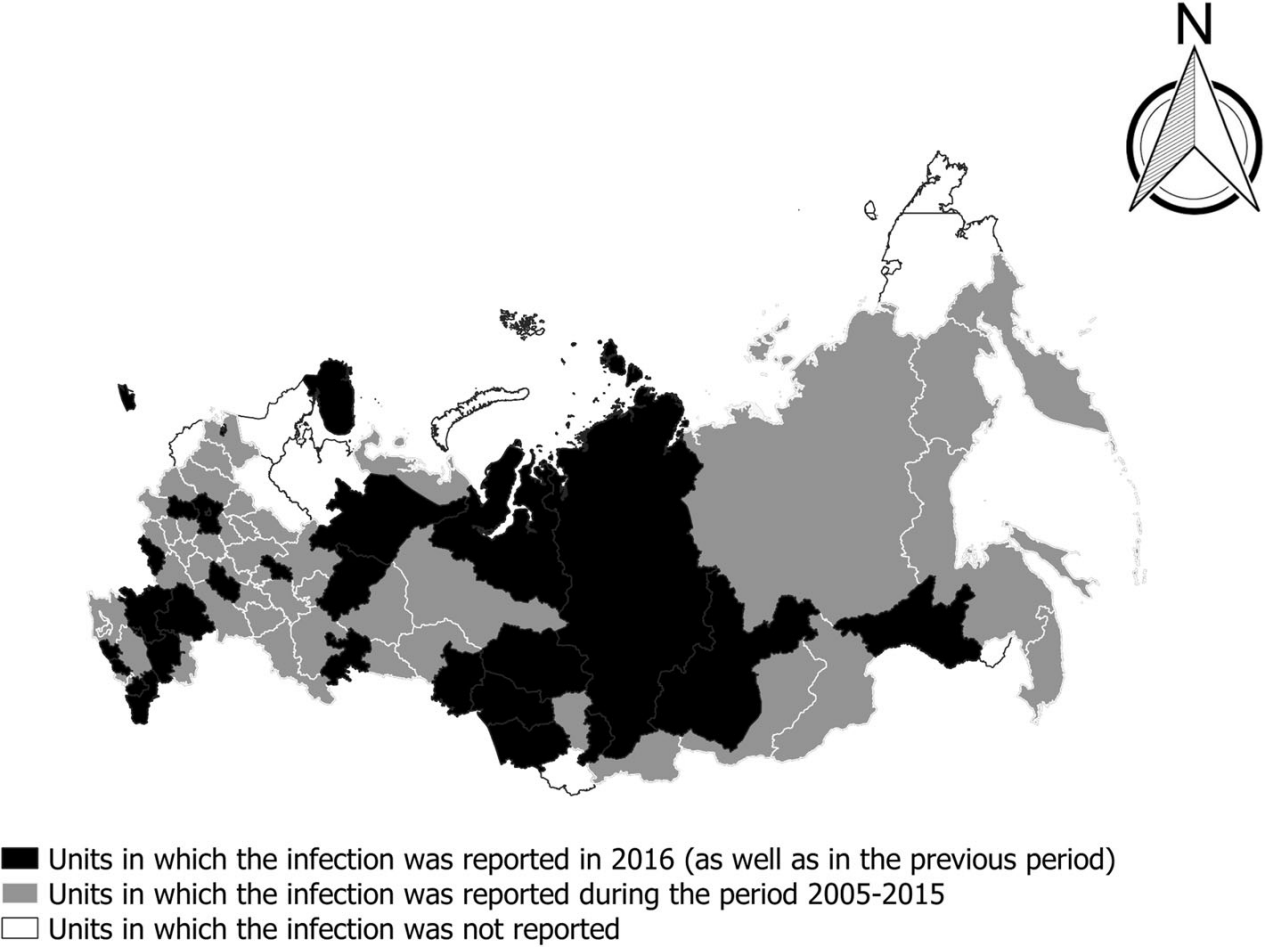
Administrative units of the Russian Federation in which *Taenia saginata* taeniosis was reported for the period 2005–2016

**Fig. 4:**
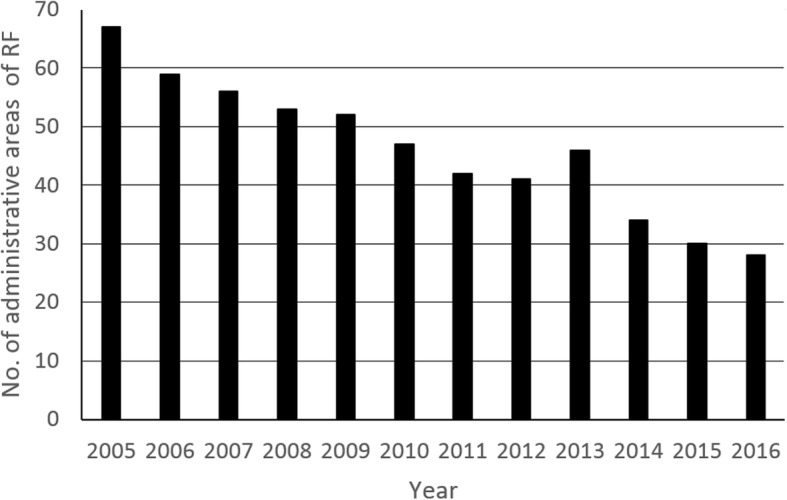
Number of administrative units of the Russian Federation in which cases of *Taenia saginata* taeniosis were registered (2005–2016)

A reduction in the infection burden is not only evident in the decline of the incidence of infection, but also in the reduction of the territory in which the infection was registered (Fig. 3). Russia formerly consisted of 85 federal subjects, which are now constituent members of the RF. During the period from 2005–2016, taeniosis cases were registered in the territory of 79 administrative units, but their number decreased yearly [17]. Thus, the number of administrative units in which the infection was registered dropped from 67 in 2005 to 28 in 2016 [17] (Fig. 4).

It is important to note that the decline in the incidence of infection, as an overall trend, has been observed in all administrative units. For instance, between 2005 and 2016, the incidence of infection in the Chechen Republic decreased from 8.1 to 0.31/100,000, in the Komi Republic from 1.7 to 0.2/100,000, in the Rostov District from 0.4 to 0.2/100,000 and in St. Petersburg from 0.04 to 0.02/100,000 [17]. Only sporadic cases have been reported in the majority of the other administrative areas. In the period from 2008 to 2016, less than 3 cases of infection were annually reported in 46% (35/75) of the administrative units [17]. The incidence of infection was significantly higher in the Asian part of the RF, compared to the European part (unpublished observations). Cases were concentrated in western Siberia, in the Yamalo-Nenets Autonomous District (in 2016 1.68 cases per 100,000 inhabitants) and in the North Caucasian Republics: Chechnya (in 2016 0.31 cases per 100,000 inhabitants), Dagestan (in 2016 0.21 cases per 100,000 inhabitants) and Kabardino-Balkar [17]; these territories are inhabited by less than 1% of the total population of RF [18].

The incidence decline is not a continuous process, so no new cases in several years in some regions do not necessarily indicate cases will not appear again. Indeed, in the Tambovska District, cases were registered in 2005, 2006, 2011, 2012 and 2014 [17].

Official data from 2005 to 2016 [9, 13–16] showed that the infection was most commonly diagnosed (*c.*70%) in patients seeking medical care. The remaining cases were detected during obligatory occupational health checks, such as sanitary control (around 28%), and 2% on the basis of epidemiological indications (epidemiological surveillance after diagnosing cases in the surroundings). The infection was more commonly diagnosed in females and more often in adults (most often in ages between 30–49). Children aged up to 5 years-old accounted for 3–5% of all diagnosed cases [9, 13–16].

Generally, the largest number of cases (60–69%) was registered in the summer, specifically in the month of August [9, 13–16]. However, that was not the case in all areas; the Chechen Republic, for example, had the largest number of cases registered between November and February (data for the period between 2004–2010) [19, 20].

Over the whole observed period among the infected individuals, the proportion of residents of urban areas increased from 46% in 2004 to 72% in 2016 [9, 13–16]. Official data showed that 40–43% of the infected were unemployed and retirees, while students accounted for 17% [9, 13–16].

Beef was a source of infection for 85–93% of the infected people [9, 13–15]. According to the origin of the meat, personal back yard slaughtering accounted for about 28–43% of all cases, while meat bought at the “farmers market” accounted for 39–47% of the cases and was thus the most common source of infection [9, 13–16]. Meat purchased in retail stores was a source of infection in only about 4–12% of cases. The most- to least-likely methods of preparing the meat for human consumption which resulted in infection were the following: raw minced meat for 44–46%, barbecue for 16–17%, cutlets for 6–7%, dried meat for 4–6%, cooked for 3–5%, roasted for 1–5%, and other dishes for 8% of the cases. Only 6–9% of the infected individuals were cattle breeders, reindeer herders, and catering workers, all of who have a professional risk of spreading the infection [9, 13–15].

### Infection in cattle

**Fig. 5:**
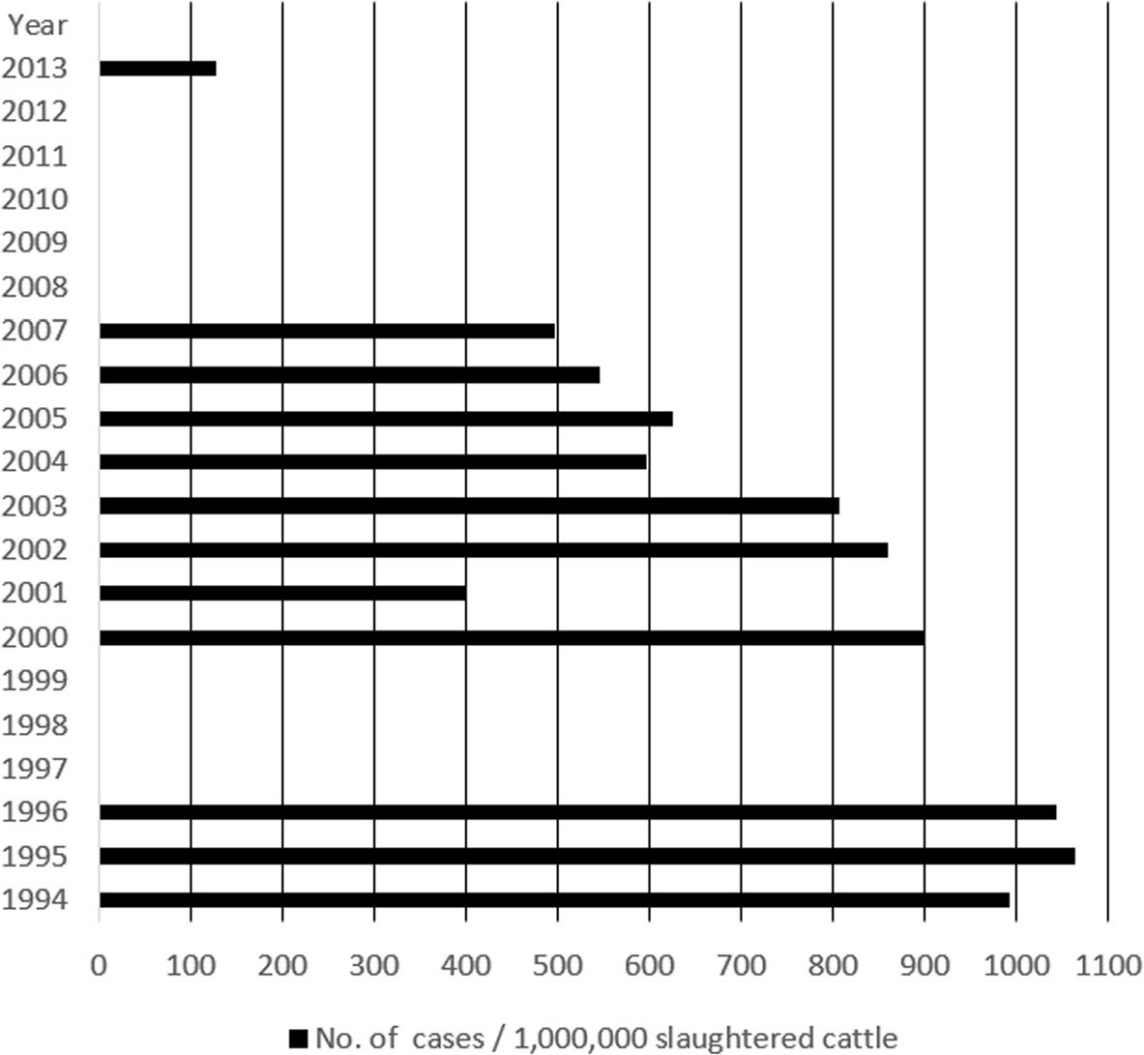
Number of bovine cysticercosis cases per 1,000,000 slaughtered cattle in the Russian Federation (1994–2016) (data for 1997, 1999 and 2008–2012 not available)

Based on annual data on the number of infected slaughtered cattle [9, 13–15, 21] and total number of slaughtered cattle [22, 23], we calculated number of infected per 1,000,000 cattle for each year (Fig. 5). General decline in infected cattle was observed, especially after 2005, but the downward trend seems less strong than in human infections.

**Fig. 6:**
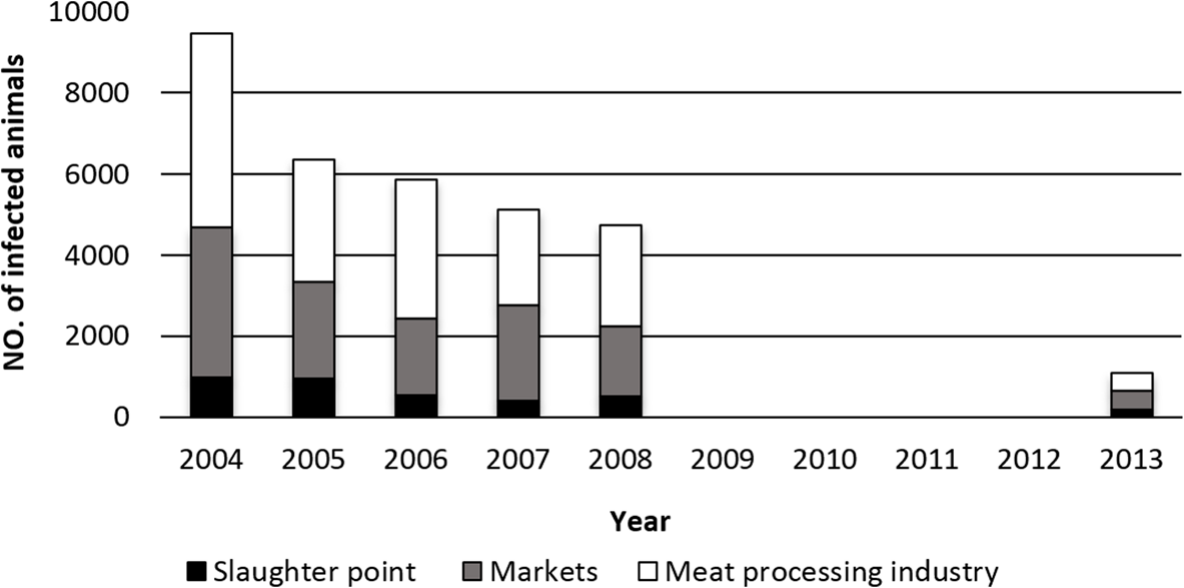
Number of infected cattle according to inspection checkpoints in the Russian Federation (data for 2009–2012 not available)

As previously mentioned, in the RF inspection of meat is carried out at three points: the slaughterhouse, meat processing plants and markets [10]. In the study period, most reports of infected cattle meat originated from meat processing plants (in the period between 2004–2008, they represented 47–58% of all reported cases) while the least number of reports came from slaughterhouses (8–15%) [9, 13–15]. A decline in the number of infected animals was present at all three check points (Fig. 6)

**Fig. 7:**
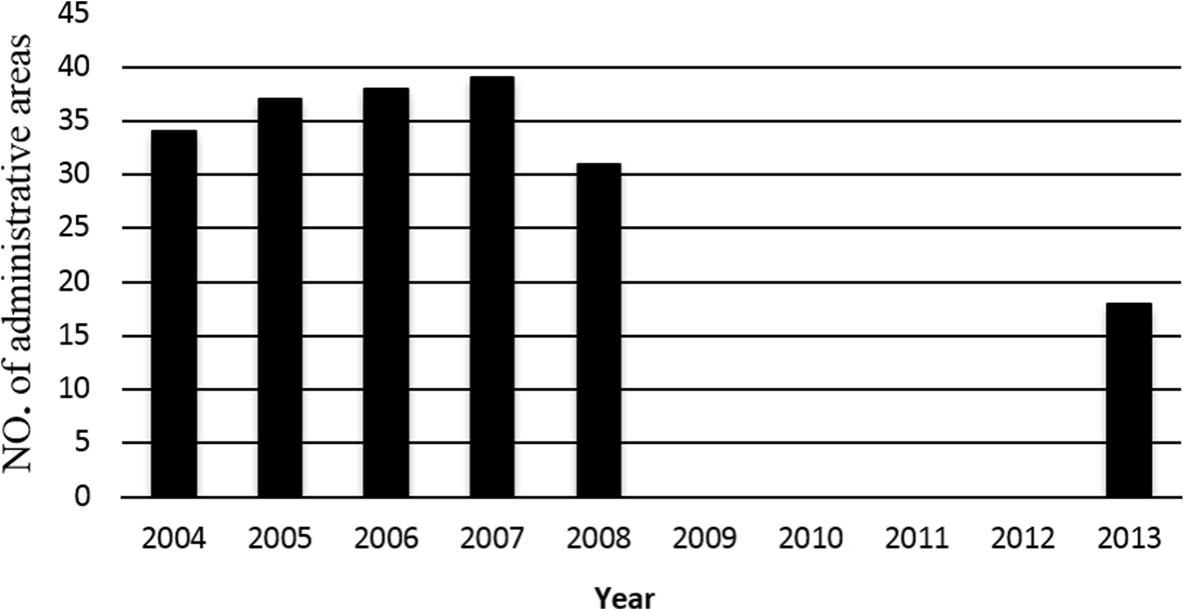
Number of Russian Federation units where infected cattle were registered in slaughtered sites (data for 2009–2012 not available)

Unlike human infection, where there was a clear reduction in the number of territories where the infection was registered, data from slaughter sites do not mirror this phenomenon [9, 13–15] (Fig. 7).

**Fig. 8:**
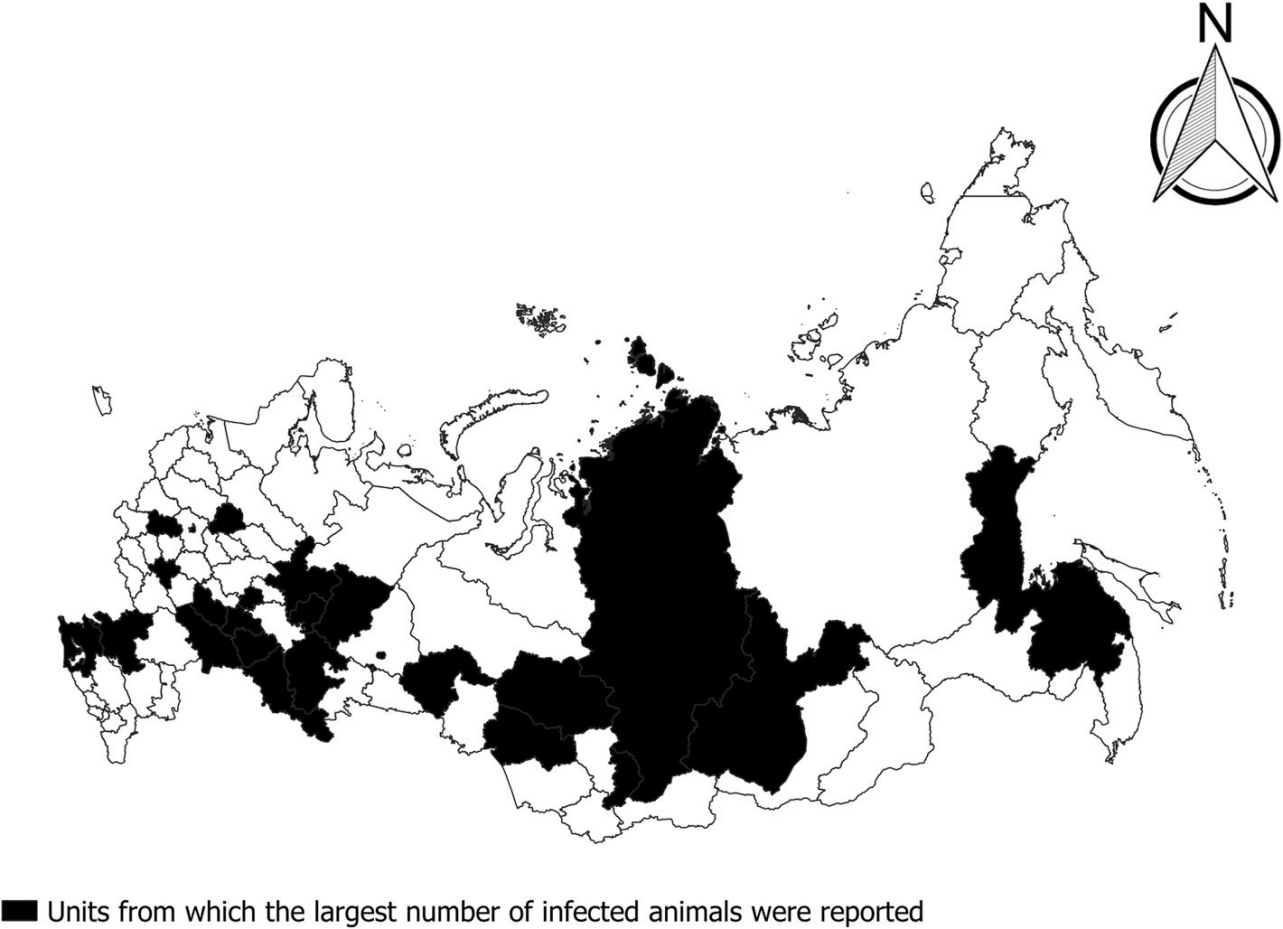
The administrative units from which the largest number of infected animals were reported (2004–2008, 2013). Official data from slaughter point and markets

Data from markets show a similar picture, but the origin of the meat cannot be precisely determined, as it is possible that it originates from neighboring areas. Cattle meat in Moscow markets is mainly from neighboring central regions of Russia (Voronezh, Lipetsk, Tambov, Penza, Kursk, Smolensk and Tver region) [24]. For this reason, in order to gain insight into the geographical distribution of infection, data from slaughter points and from markets in the areas from which the largest number of infected animals were reported in the period between 2006–2013 [9, 13–15] are presented together (Fig. 8). The geographical distribution of the areas from which most of the infected animals have been reported are partially in-line with the distribution of cattle breeding. In 2015, the largest numbers of cattle were concentrated in five regions: the Altai Territory and the republics of Bashkortostan, Dagestan, Tatarstan, with the greatest density in the Republic of Ingushetia, from 31–65 head of cattle per km^2^ [25].

It is interesting to note there is no constant trend of change in the infection prevalence in cattle from the same territory. In the Province of Altai [9, 13–15], in the period between 2000–2011, the maximum level of infection, according to data from slaughterhouses, occurred in 2002 (0.061%). In subsequent years, the percentage of bovine cysticercosis cases declined, and in 2006 and 2007 no cysticerci were detected. However, the infection reappeared later on, and the prevalence reached 0.031% in 2011. In some areas, such as the Middle Ural, along the Kama River [26], mutually adjacent zones have a high (0.5–5%), average (0.3%) and low (< 0.1%) infection levels, indicating a mosaic distribution of cysticercosis in cattle.

### Infection in reindeers and yaks

In addition to beef, local reindeer are an additional source of human infection in Russia (7–15% of all cases) [9, 13–15]. For the “northern strain” of *T. saginata* that occurs in the RF, reindeer are the intermediate host [27]. Domesticated reindeer play an important role as a source of infection among the Nenets, Komi, Khanty and other native ethnic groups living in northern Russia (Yamalo-Nenets Autonomous District and Nenets Autonomous District Komi Republic Khanty-Mansi Autonomous District), in whose cuisine fresh, raw brain, or “*stroganina*” (thin cuts of frozen fresh meat), or steamed and dried meats are traditionally consumed [28]. In the Yamal-Nenets Autonomous District, according to the epidemiological investigation, all cases of *Taenia* spp. infection reported in 2015 occurred as a result of consumption of raw reindeer brain (uncontrolled slaughtering) [29].

Domestic yaks (North Caucasian ecotype) in Russia live in the territory of the North Caucasus republics, in Kabardino-Balkaria, Karachaevo-Cherkessia, Dagestan, Checheno-Ingushetia and North Ossetia, where domesticated varieties represent a highly valuable source of meat. The prevalence of infection in yaks is high, at 3.4% [30].

## Discussion

In the RF, *T. saginata* infection is notifiable for both humans and animals. A relatively small number of published papers were included in the present analysis, as most of the publications, especially those related to particular administrative units of the RF, rely on data from official reports, which were already included in the analysis.

For prevalence of *T. saginata* in humans, data were taken from official reports, which report *T. saginata* and *T. solium* infection separately, not questioning their accuracy. As microscopic examination of feces samples was the basis for diagnosis (morphological distinction between *T. saginata* and *T. solium* is difficult in routine examination), the reported number of *T. saginata* taeniosis cases may not absolutely reflect the actual situation. However, as the frequency of *T. saginata* infection is significantly higher than *T. solium* in the RF (in 2014, 102 cases of *T. saginata* taeniosis and 42 of *T. solium* were registered) [17], it may not have a great impact on the accuracy of the data.

It is clear that the reduction in the incidence of infection over the period of interest is a continuation of a long-term decreasing trend that has been driven by constant monitoring and the implementation of preventive measures since the 1960’s, and this reduction is also reflected in the incidence of *T. soluim* infection [9, 13–17]. Over the past 57 years, from 1960 to 2016, the incidence of *T. saginata* taeniosis in the RF has decreased from 46.06 to 0.04/100,000 inhabitants. After 1991, a rapid decline of infection incidence could potentially be a consequence of a decline in cattle and reindeer breeding between 1991–2017 (3-fold decline between 1991 and 2006) [23, 31]. In response to this decline in local production, there has been an increased consumption of imported meat, which is mostly transported long enough at low temperatures to kill *Cysticercus bovis* cysterici and is repeatedly controlled [19].

In the period between 1987 and 1994, taeniosis was registered in all 85 administrative units of the RF. Of those, 69 (81%) had a mean incidence of infection from 0.09–4.9/100,000 inhabitants [32]. During the period between 2005–2016, taeniosis was registered in 79 units, only 28 in 2016, with an incidence of less than 0.09/100,000 inhabitants in 20 units and more than 1/100,000 only in the territory of the Yamalo-Nenets Autonomous District. The occurrence of sporadic cases in a large number of areas is probably the result of traveling or immigration from endemic areas, or consumption of meat coming from endemic regions rather than local transmission. In 2012, cases imported from Chechnya, Dagestan, Armenia and Tajikistan were registered in Moscow [33]. Such sporadic cases can of course lead to contamination of the environment and completion of the life-cycle, with increased infection in cattle.

Given the vastness of the Federation and all the differences and specificities of particular areas (climate, level of development, population, etc.) it is not surprising that the rate of decline varies among the regions. Meat consumption is traditionally very different by regions; for instance, in northern Russia, while Murmansk and Arkhangelsk are areas with low meat consumption (< 70% of recommended quantities), the people of the neighboring Yamalo-Nenets area are higher meat consumers (> 95% of recommended quantities) [34].

Differences observed in infection incidences based on the season can be attributed to traditional periods for slaughtering and differences between common summer and winter dishes. In northeast areas, such as Yakutia, fresh meat is eaten mostly in the summer and early fall. During the summer months, the urban population also contributes partly to the increase in the number of cases by outdoor food consumption, and buying meat for grilling in local households or farmers markets. In the southern regions, the late autumn and winter are traditional times for slaughtering and fresh meat consumption [33].

Even today, the role of extensive livestock farming is important, and livestock distribution depends on extensive pastures. Areas with a long standing livestock rearing and tending tradition have always been areas where it is difficult to implement the necessary hygiene and sanitary conditions as well as meat inspection, especially if the meat is meant for personal use. Areas in which the population traditionally consumes insufficiently cooked or improperly cured meat, such as in the northern regions, where reindeer brain, or “*stroganina*” (frozen fresh meat) eaten and Caucasia where “ *shashlik*” (a dish of skewered and grilled cubes of meat) are eaten, and central Asia with “*basturma*” (cured meat) and “*bichak*” (dough filled with ground meat), and Transbaikalia with “ *buuz*” (dough filled with ground meat), are also areas with a higher prevalence of infection. Another factor may be the developing urbanization, whereby areas of livestock breeding find themselves closer to the cities. In the Caucasus, the high level of environmental pollution by *Taenia* spp. eggs in areas of frequent human traffic and poor sanitary conditions (such as city suburbs, picnic areas or rest areas) [35] singles them out as new hot spots for the infection of the local livestock breeding areas and/or hay production in the surrounding meadows. This is especially important because uninspected meat from these areas easily arrives at the unofficial market where no control is practiced.

In the spread of *T. saginata*, backyard slaughtering with no meat inspection has, if not the most important, a very important role. In 2016, 29% of cases were infected with meat from backyard slaughtering of cattle raised on their own farmstead [16]. This meat also finds its way to the unofficial markets, which may explain the social structure of the infected people. Most often they are retirees and the unemployed, those who consume meat purchased from cheaper uncontrolled markets. This also explains why the urban population is more frequently infected than rural inhabitants. Another reason could be migration to cities, but this has risen the number of urban residents in the RF for only 4.4% between 1993 and 2006 [36].

The frequency of bovine cysticercosis in Russia is generally declining, especially after 2005. Data on animal infection in official records were less accessible and detailed than the data regarding human infections. The lowest numbers of infected animals were reported from the places of slaughter, and significantly more after inspection at the markets. This can be partly explained by insufficient sensitivity of the visual inspection method. At a primary inspection of 1145 beef hearts, *T. saginata* cysts were found in 1.3% of cases, but re-inspection revealed another 2.3% cases [37]. Lack of inspection, especially for backyard slaughtering, is an important part of the explanation too. In 1997, 56% of cattle were slaughtered on farms and this trend continued in the following years [7].

It is reasonable to suspect that the data from places of slaughter underestimate the real number of infections. However, since the reasons for underestimation have been consistently present, the declining trend of infection seems beyond suspicion. This is also confirmed by the reduction in the number of infected animals in industrial plants where the meat comes from various regions, and the conditions of inspection are better.

Decrease of *T. saginata* infection in humans and cattle in the RF confirms the effectiveness of this approach to prevention. Some omissions in implementation of control strategies do reduce the effectiveness somewhat, the most important probably being insufficient coverage of the slaughter points by control, and inefficiency of the reporting system. Official reports also mention the need for better control of sanitary cleaning of urban and rural settlements, and development and introduction of effective methods for disinfection of sewage and also better interaction of sanitary and epidemiological experts, veterinary supervisory authorities and law enforcement agencies. It should also be expected that the revitalization of livestock breeding, which is currently underway in Russia [38], through investments in the modernization of existing and the construction of new breeding capacities, will lead to a further reduction in the infection rate of the cattle. At the same time, the tendency of increasing the participation of small farms in cattle breeding and reducing the participation of large agribusinesses makes control more complex [31].

## Conclusions

As a result of several decades of effort against *T. saginata*, the importance of this parasitic infection as a medical and veterinary problem decreased in the 21st century in the RF. Importantly, the previously well-established prevention systems continued to function even through the changes after 1991. However, *T. saginata* taeniosis/cysticercosis remains a health and economic impact in the RF. The continuous existence of focal points of infection, as well as cyclical disappearance and re-emergence of infection outside the established foci, imposes the need for further surveillance and application of preventive measures, especially more comprehensive control of meat.

## Abbreviations

PRISMA: Preferred Reporting Items for Systematic Reviews and Meta-Analyses
RF: Russian Federation
USSR: Union of Soviet Socialist Republics
